# Proton-Conducting Polymeric Membranes Based on 1,2,4-Triazole

**DOI:** 10.3390/membranes13020169

**Published:** 2023-01-29

**Authors:** Galina F. Prozorova, Alexander S. Pozdnyakov

**Affiliations:** A.E. Favorsky Irkutsk Institute of Chemistry, Siberian Branch, Russian Academy of Sciences, 664033 Irkutsk, Russia

**Keywords:** proton-conducting membranes, 1-vinyl-1,2,4-triazole, proton conductivity

## Abstract

In this review, a comparative analysis of the literature and our own results obtained in the study of the physicochemical, dielectric, and proton-conducting properties of composite polymer materials based on 1*H*-1,2,4-triazole has been carried out. It has been established that 1*H*-1,2,4-triazole and homopolymers and copolymers of 1-vinyl-1,2,4-triazole are promising for the development of proton-conducting fuel cell membranes. They significantly improve the basic characteristics of electrolyte membranes, increase their film-forming ability, increase thermal stability up to 300–330 °C, increase the electrochemical stability region up to 3–4 V, promote high mechanical strength and morphological stability of membranes, and provide high ionic conductivity (up to 10^−3^–10^−1^ S/cm) under anhydrous conditions at temperatures above 100 °C. There is also an improvement in the solubility and a decrease in the glass transition temperature of polymers based on 1-vinyl-1,2,4-triazole, which facilitates the processing and formation of membrane films. The results obtained demonstrate the uniqueness of 1*H*-1,2,4-triazole and (co)polymers based on 1-vinyl-1,2,4-triazole and the promise of their use for the creation of heat-resistant plastic and electrochemically stable, mechanically strong proton-conducting membranes with high ionic conductivity under anhydrous conditions and at high temperatures.

## 1. Introduction

The development of proton-conducting fuel cell membranes is an urgent task due to the high demand for efficient power sources in the rapidly developing industries of portable electric appliances, electric vehicles, spacecrafts, submarines, etc. Currently, fuel cells are the most versatile and popular sources of energy. They are characterized by high efficiency, fast start-up and dynamic responses, and small dimensions. Proton-conducting membranes are the main components of fuel cells. The main role of proton-conducting membranes is to ensure proton transport and the efficient separation of electrodes in order to prevent their electrical contact, as well as the direct chemical reaction of molecular reagents.

The studied polymeric proton-conducting membranes consist mainly of hydrophobic fragments (fluorocarbon and ether chains) and hydrophilic phases (acidic sulfo- and phosphorus groups). Hydrophilic fragments of polymers provide efficient proton transfer, while hydrophobic groups contribute to high mechanical strength and morphological stability of the membranes. Water, reacting with the acidic groups of the polymer, forms nanosized hydrated clusters, which dissociate to form hydrated mobile protons.

There are several types of electrolyte membranes used in the development of fuel cells. The main ones are perfluorinated sulphate-containing electrolyte membranes, such as “Nafion” (E.I. Du Pont de Nemours Co. Inc., Wilmington, Delaware, USA), “Flemion” (Asahi Glass Company, Tokyo, Japan), “Neosepta-F” (Tokuyama Soda Co. Ltd., Tokyo, Japan), and MF-4SK (OAO Plastpolimer, St. Petersburg, Russia), based on copolymers of tetrafluoroethylene with perfluorinated sulfonic monomers [1,2,3,4,5,6,7,8,9,10]. These membranes are characterized by high proton conductivity (up to 10^−1^ S/cm) at high humidity and temperatures up to 80 °C, as well as good mechanical characteristics; however, the use of such membranes in medium-temperature fuel cells is limited due to their low ionic conductivity at low humidity and elevated temperatures, their tendency toward destruction, and their high cost.

Therefore, intensive research on the development of polymeric proton-conducting membranes for fuel cells with commercial availability and high-performance characteristics (proton conductivity, chemical stability) in a wide temperature range (100–200 °C) continues. Of particular interest are proton-conducting membranes based on polymers with fluorinated and sulfonated styrene fragments [11,12]. However, the conductivity of such membranes decreases during long-term use due to a number of destructive processes, particularly acid leaching from the membrane volume, which limits the possibility of their practical use.

To fabricate proton-conducting membranes, heteropoly compounds and various polymers containing active pyridine fragments, SO_2_, CH_3_, SO_3_Na, and OH groups in macromolecules, which provide high proton conductivity (up to 10^−1^ S/cm) in a wide frequency range, are studied, examining temperature range (90–170 °C), mechanical strength, and chemical stability [13]. However, the strong dependence of the conductivity of these membranes on the relative humidity of the environment limits their use in fuel cells.

A large amount of research is devoted to the development and study of proton-conducting membranes based on polybenzimidazole (PBI) and its derivatives, which are characterized by high proton conductivity [14,15,16,17,18,19,20]. It has been shown that the modification of PBI by sulfonation and phosphorylation leads to a significant improvement in the proton-conducting, mechanical, and thermal characteristics of PBI-based membranes. It was assumed that the use of imidazole in the production of electrolytes would provide intermolecular proton transfer at temperatures above 100 °C and the corresponding performance of new-generation fuel cells. However, the low solubility, high glass transition temperature (300–400 °C), and insufficient electrochemical stability of imidazole, due to the high electron density of the imidazole ring, prevent the widespread use of PBI for the manufacturing of membranes [21,22].

**Figure 1:**
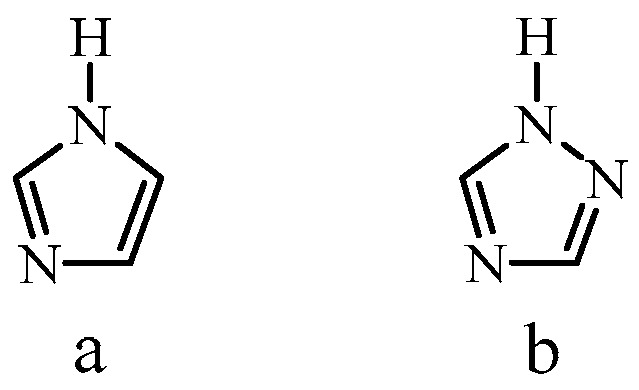
Molecular structure of imidazole (**a**) and 1*H*-1,2,4-triazole (**b**).

The molecule 1*H*-1,2,4-triazole, which has a molecular structure close to that of imidazole, has better electrochemical stability compared to imidazole [23] (Figure 1).

The proton conductivity of pure 1*H*-1,2,4-triazole is 1.5 × 10^−4^ S/cm at 115 °C and 1.2 × 10^−3^ S/cm at a melting point of 120 °C [23]. The mechanism of proton conductivity of 1*H*-1,2,4-triazole is similar to that of imidazole and consists in the intermolecular transfer of protons (structural diffusion) [23]. At the same time, due to the presence of single–double alternating bonds and three nitrogen atoms in the triazole ring, proton transfer in triazole is easier than in imidazole. There is also an improvement in solubility and a decrease in the glass transition temperature of triazole-based polymers, which increases the possibility of processing and forming membrane films [23]. Along with this, 1*H*-1,2,4-triazole is characterized by a higher chemical stability and a higher melting point (120 °C). It was noted that the use of 1*H*-1,2,4-triazole for the modification of polymer layers leads to an improvement in the characteristics of proton-conducting membranes based on them and an increase in the proton conductivity of membranes at temperatures above 100 °C [23,24,25,26,27,28,29,30,31,32].

Thus, in the formation of polymeric proton-conducting membranes, the main role of the polymer is to ensure the thermal and mechanical stability and plasticity of the membrane matrix. Doping polymer membranes with various acids (H_3_PO_4_, CF_3_SO_3_H, CH_3_SO_3_H, etc.) leads to an increase in the proton concentration. Additionally, modification of the polymer matrix of membranes with 1*H*-1,2,4-triazole promotes an increase in proton transfer due to the coordination interaction of triazole fragments with acid and the formation of low-barrier hydrogen bonds between the molecules of 1,2,4-triazole and acid [23,24,26].

The main results of the studies demonstrating the positive effect of 1*H*-1,2,4-triazole on membrane characteristics are presented in Table 1.

This review analyzes the results from studies of the electrical properties and efficiency of using 1-vinyl-1,2,4-triazole homo- and copolymers for the development of proton-conducting fuel cell membranes.

## 2. Physicochemical and Electrophysical Properties of Poly-1-Vinyl-1,2,4-triazole

**Figure 2:**
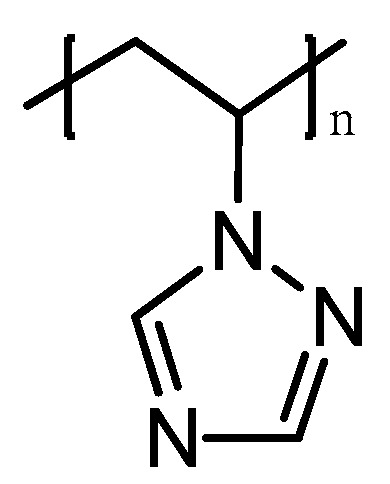
Molecular structure of poly-1-vinyl-1,2,4-triazole.

Poly-1-vinyl-1,2,4-triazole (PVT) is a unique polymer with a wide range of valuable, practically significant properties (Figure 2).

Polymers and copolymers of 1-vinyl-1,2,4-triazole are characterized by water solubility, nontoxicity (LD_50_ > 5000 mg/kg), chemical resistance, high thermal stability (up to 300–350 °C), complex formation, sorption and biological activity, and high stabilizing ability in the formation of nanoparticles of various metals (Ag, Au, Cu, Fe) [33,34,35,36,37,38,39,40,41,42].

PVT exhibits the properties of organic dielectrics and is characterized by a specific electrical conductivity at room temperature of the order of 10^−15^–10^−16^ S/cm and an activation energy of 2.1–3.2 eV, depending on the molecular weight [43]. PVT is a promising dielectric for the development of organic field-effect transistors (OFETs) [44]. It provides good film-forming properties, low leakage current, and fairly high breakdown voltage. [44]. The energy structure and nature of conduction in PVT have been studied by the methods of charge-limited currents, thermally stimulated depolarization, and photoinjection currents [45,46].

The possibility of obtaining structurally ordered systems based on PVT using structured water as a solvent has been demonstrated [47]. The electronic properties of films obtained from a solution of PVT in structured water are similar to crystalline samples, while films obtained from solutions of PVT in distilled water have an amorphous structure [47].

It has been reported that nanocomposites based on PVT with Fe_3_O_4_ nanoparticles are characterized by electrical conductivity on the order of 10^−4^–10^−6^ S/cm in an alternating current field [48]. One-pot synthesis of nanocomposites with BaFe_12_O_19_ nanoparticles in a PVT matrix was carried out [49,50]. It was shown that the ionic conductivity of the nanocomposites was 10^−7^–10^−12^ S/cm as a function of the frequency of the alternating current. Using emulsion polymerization, PVT nanocomposites with single-walled nanotubes (SWNT) [51] and multiwalled carbon nanotubes (MWCNT) [52] were synthesized, which demonstrated good solubility, had an electrical conductivity of about 10^−2^ S/cm, and were promising for use in fuel cells. Upon copolymerization of 1-vinyl-1,2,4-triazole with N-vinyl-2-phenylpyrrole and N-vinyl-4,5,6,7-tetrahydroindole, the electrical conductivity of the samples was 10^−14^ S/cm [53,54]. After doping the copolymers with iodine, the electrical conductivity increased to 10^–7^ S/cm [54].

## 3. Proton-Conducting Membranes Based on 1-Vinyl-1,2,4-triazole Homopolymers

A large amount of research is devoted to the development and study of proton-conducting membranes based on 1-vinyl-1,2,4-triazole homopolymers (Table 2).

In the studies [55,56,57,58], poly-1-vinyl-1,2,4-triazole (PVT) was produced by free radical polymerization of 1-vinyl-1,2,4-triazole and was doped with phosphoric acid and nitrilotri(methyl-triphosphonic acid) at various molar ratios. The synthesized PVT is thermally stable up to approximately 250 °C. The proton exchange reactions between PVT and H_3_PO_4_ (or C_3_H_18_NO_24_P_9_) was proved with Fourier transform infrared spectroscopy. After doping PVT, the intensities of the bands corresponding to the triazole ring stretching (C–N, C=N) vibrations between 1430 and 1650 cm^−1^ changed [56]. Additionally, a strong peak at 3100 cm^−1^ could be associated with N–H absorption in the protonated triazole. A broadening of the band between 3500 and 2000 could be related to a hydrogen bonding network formation. Within the 1800–900 cm^−1^ region, the peaks near 1100 cm^−1^ and 979 cm^−1^ were attributed to characteristic absorptions of the HPO_4_^2−^ and H_2_PO_4_^−^ in the blends [56]. The presence of HPO_4_^2−^ and H_2_PO_4_^−^ anions implied that phosphoric acid is ionized in the blend system. The proton conductivity of these materials increased with dopant concentration and temperature. In the anhydrous state, the proton conductivity of PVT—H_3_PO_4_ was 5.0 × 10^−3^ S/cm at 150 °C [56] and the conductivity of PVT—C_3_H_18_NO_24_P_9_ was 8.5 × 10^−4^ S/cm at 150 °C [57,58].

In the work [59], the results of a study of PVT complexes with poly(vinylphosphonic acid) (PVPA) are discussed. The complexes of various compositions were synthesized and homogeneous plastic materials were formed on their bases. The positive effect of PVT was its ability to prevent the formation of phosphonic acid anhydrides up to 150 °C. The PVT-PVP-based membrane showed a proton conductivity of 2.5 × 10^−5^ S/cm at 180 °C in the anhydrous state. After humidification (RH = 50%), PVT-PVPA showed a proton conductivity of 0.022 S/cm at 100 °C, which is close to that of Nafion 117 at the same humidity level [59].

Homogeneous proton-conducting membranes, thermally stable up to 250 °C, were synthesized by complexation of PVT with poly(styrene sulfonic acid) [60]. The maximum proton conductivity of the obtained membranes in the anhydrous state was 0.033 S/cm at 120 °C [60].

Membranes obtained based on PVT doped with toluenesulfonic acid at various molar ratios (0.5, 1, 1.5, 2, with respect to the polymer repeating unit) are characterized by thermal stability up to 250 °C, electrochemical stability over 3 V, and proton conductivity of 8.0 × 10^−4^ S/cm at 150 °C and 0.012 S/cm at 110 °C [61].

Promising membranes were obtained by doping PVT with trifluoromethanesulfonic (triflic) acid (CF_3_SO_3_H) at several molar percentages (25, 50, 75, 100, and 150%) with respect to the polymer repeat unit [62]. Due to the strong interaction between of sulfonic acid groups of triflic and the triazole units of PVT, no phase separation occurred during solvent evaporation; hence homogeneous and transparent films formed. The thermal stability of these membranes was 300 °C, depending on the acid content. Maximum proton conductivity of 0.012 S/cm at 80 °C was obtained for PVT doped by triflic acid, which is comparable to that of hydrated Nafion [62].

New proton-conducting materials, such as transparent thin films, were obtained by the complexation of PVT and poly(2-acrylamido2-methyl-1-propanesulfonic acid) at various ratios (from 0.25 to 4) [63]. The maximum values of proton conductivity were obtained for the complexes of PVT and acid with ratio compositions 1:2 and 1:4. In the anhydrous state, the proton conductivities of these complexes at 150 °C were 1.1 × 10^−8^ S/cm and 1.2 × 10^−6^ S/cm, respectively. The proton conductivities of these complexes in the hydrated state increased significantly, namely, to 0.3 S/cm and 0.06 S/cm, respectively, at 100 °C [63].

Polymer–polymer blends based on PVT are promising alternative proton-conducting materials. In study [64], a simple and economical method for obtaining new polymeric materials was carried out by mixing two polymers with different physical properties (PVT and polybenzimidazole (PBI)) in order to obtain a material with improved physical and chemical properties, namely high proton conductivity and high thermal and mechanical stability. The authors [64] carefully analyzed the FT-IR spectra of PVT, PBI, and blend films in the range of 1700–1150 cm^−1^ and found that there was an N–H ··N type of interaction between the two polymers. This confirmed the formation of hydrogen bonds between the two polymers in the mixture. The presence of interactions between the two polymers was also confirmed by the results of solid-state NMR, in which a shift of the characteristic PBI peaks to a higher field in a mixture of polymers was observed, as well as by corresponding changes in the absorption and emission spectra of the fluorescence of the PBI and PVT samples, and that of their mixture [64]. All samples of the mixture in their unalloyed state were thermally stable up to 300 °C. A significant increase in the proton conductivity of the polymer mixture doped with phosphoric acid was found. The maximum values of proton conductivity were observed for a 50:50 mixture of polymers equal to 1.1 × 10^−1^ S/cm at 160 °C, which is an order of magnitude higher compared to pure PBI. The main reason for the high proton conductivity of mixed membranes is their more porous morphology, which contributes to a higher degree of doping with phosphoric acid, which leads to an increase in proton conductivity.

New types of composite membranes were synthesized by crosslinking poly(vinyl alcohol) (PVA) with sulfosuccinic acid (SSA) and intercalating PVT into the resulting matrix [65]. The resulting hybrid membranes were transparent, flexible, heat-resistant up to 200 °C, and had a proton conductivity of (1.6–7.7) × 10^−5^ S/cm at 150 °C. After wetting the membranes (RH=100%), the proton conductivity increased to 2.8 × 10^−3^ S/cm at 60 °C [65]. An increase in the absorption of the solvent (water/methanol) was established with an increase in the content of PVT in the membranes.

**Figure 3:**
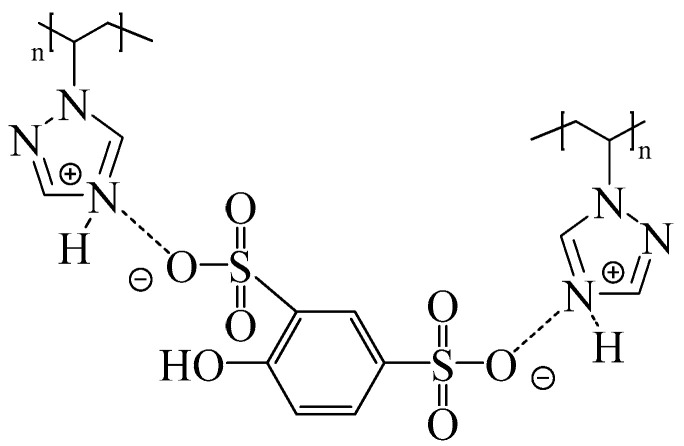
Structure of PVT–PDSA acid–base complexes.

We recently reported on the development of new proton exchange membranes based on PVT and phenol-2,4-disulfonic acid (PDSA) with different ratios, as well as the additional use of polyvinyl alcohol crosslinked with oxalic acid [66,67]. It has been shown that due to the transfer of protons from PDSA to the triazole fragments of PVT, acid–base complexes are formed, which contribute to an increase in the mechanical and thermal stability (up to 245 °C of the resulting membranes) (Figure 3). Furthermore, ^15^N NMR proves that the PVT triazole rings in the membranes are protonated by PDSA.

The proton conductivity of the membranes and the activation energy of proton transfer increased with an increase in the content of PDSA in their composition and amounted to 6.0 × 10^−2^, 7.3 × 10^−3^, and 6.2 × 10^−3^ S/cm (at a temperature of 80 °C and a humidity of 75%) and 19.5, 21.6, and 38.2 kJ/mol for membranes, containing 36, 24, and 8 mass.% of PDSA, respectively [66,67].

The proton-conducting polymeric membranes were obtained by mixing PVT with sulfonated polysulfone and phosphoric acid [68]. In the course of the reaction, an interaction took place between the triazole fragments and sulfonic acid units, which was confirmed by the data from the Fourier transform infrared spectroscopy. Proton exchange reactions between the polymer and H_3_PO_4_ were established. The obtained membranes were thermally stable up to 150 °C and possessed a maximum proton conductivity of 3.63 × 10^−4^ S/cm at 150 °C [68].

The originality of PVT is evidenced by the results of study [69], which demonstrated that the insertion of PVT into hydrophilic Nafion channels led to the production of electrochemically, thermally, and mechanically stable membranes. The authors of [69] studied a mixture of Nafion with PVT. Due to the strong interaction between the sulfonic acid groups of Nafion and the triazole units of PVT, phase separation did not occur during solvent evaporation, and homogeneous and transparent films were formed. In a mixed system, proton conduction can occur through triazole units, which, when combined, form dynamic hydrogen chains that support long-distance proton transport. The conductivity of the Nafion-PVT-blend membranes was measured to be 5.3 × 10^−4^ S/cm at 220 °C in an anhydrous state, and increased at least three orders of magnitude upon hydration. At the same time, these Nafion-PVT membranes are characterized by high thermal stability up to 300 °C and low methanol permeability, namely two times lower than that of commercial Nafion 112 [69].

## 4. Proton-Conducting Membranes Based on 1-Vinyl-1,2,4-triazole Copolymers

Copolymerization of N-heterocyclic monomers and acidic monomers has been employed for the development of anhydrous proton-conducting membranes since N-heterocycles may act as proton solvents whereby acidic groups serve as proton-donating sites. These copolymer membranes display high proton conductivity at elevated temperatures and in anhydrous conditions. There are a number of studies on the development of proton-conducting membranes based on 1-vinyl-1,2,4-triazole copolymers (Table 3) [70,71,72,73,74,75,76,77,78,79].

It has been established that copolymers based on 1-vinyl-1,2,4-triazole with methyl methacrilate and fluoroalkylmethacrylates are able to form high-quality elastic films [70,71,72]. Composite membranes based on these copolymers are characterized by chemical stability in the face of aggressive media (acids, alkali, hydrogen peroxide), thermal stability up to 350 °C, and high moisture absorption, as well as mechanical strength. Their breaking strength is (2–7) × 10^7^ N/m^2^ and elongation is 5–7% [72]. The copolymer films doped with orthophosphoric acid are characterized by proton conductivity of about 10^−3^–10^−2^ S/cm at room temperature, with increases up to 10^−1^ S/cm with an increase in temperature up to 130 °C [70,71,72].

In the work [73], the synthesis, thermal stability, and proton conductivity of copolymers of 1-vinyl-1,2,4-triazole with vinylphosphonic acid were studied. The copolymers were synthesized by the method of radical copolymerization. As in previous works, the ability of PVT to suppress the formation of phosphonic acid anhydrides during the reaction was noted, which was confirmed by 31P NMR and TGA data. The copolymer with a triazole content of 33% had a proton conductivity of 10–3 S/cm at 120 °C in the anhydrous state, which was much higher than that of imidazole copolymers [73].

The copolymers of 1-vinyl-1,2,4-triazole with 2-acrylamido-2-methyl-1-propanesulfonic acid are characterized by high thermal (up to 250 °C) and electrochemical (more than 3 V) stability, as well as a high proton conductivity of 2 × 10^−3^ S/cm at 130 °C in the anhydrous state [74]. It has been established that proton conductivity in the copolymers is carried out according to the hopping mechanism due to structural diffusion [74].

The novel copolymers based on 1-vinyl-1,2,4-triazole and 5-(methacrylamido) tetrazole were prepared by conventional free-radical copolymerization at several monomer feed ratios [75]. The copolymer samples were doped with H_3_PO_4_ at several stoichiometric ratios to obtain proton-conductive copolymer electrolytes. The obtained membranes were thermally stable up to approximately 220 °C and structurally homogeneous. Their electrochemical stability window was 3 V. The copolymer membranes showed maximum proton conductivities of 1.6 × 10^−2^ S/cm at 150 °C and in anhydrous conditions [75].

The authors of work [76] synthesized and studied new copolymers based on 1-vinyl-1,2,4-triazole and diisopropyl-p-vinylbenzyl phosphonate. The choice of monomers was due to the fact that phosphonic acid polymers have high proton conductivity, while heterocyclic compounds improve membrane properties under anhydrous conditions. The obtained copolymers were then hydrolyzed to produce poly(vinyl triazole-co-vinyl phosphonic acid) copolymers thermally stable up to 300 °C. In order to increase the proton conductivity, the copolymers were doped with H_3_PO_4_ at several stoichiometric ratios. The proton conductivity increased with triazole and phosphoric acid content, reaching a maximum value of 0.005 S/cm at 150 °C in the absence of humidity [76].

In study [77], the high performance of poly(vinylidene fluoride) and the proton conductivity of poly(1-vinyl-1,2,4-triazole) were combined in a graft copolymer by the polymerization of 1-vinyl-1,2,4-triazole on a poly(vinylidene fluoride)-based matrix under UV light in one step. The obtained copolymers were doped with triflic acid of various concentrations, and the elastic polymer membranes were formed on their basis. The resulting membranes had high thermal stability (up to 330–390 °C) and electrochemical stability up to 4.0 V. The maximum proton conductivity of the membranes was 6.0 × 10^−3^ S/cm at 150 °C and in anhydrous conditions [77].

In study [78], novel block copolymers based on 1-vinyl-1,2,4-triazole, 1-vinyl-1,2,4-triazolium salts, and N-vinylpyrrolidone were synthesized by RAFT polymerization. Poly(1-vinyl-1,2,4-triazole) and poly(N-vinylpyrrolidone) were selected as nonionic water-soluble segments and 1-vinyl-1,2,4-triazolium salts as the ionic segment. The ionic conductivity of the obtained block copolymers was 3.1 × 10^−4^ S/cm at 90 °C [78].

Recently, the first report has appeared on the growing structure of a block copolymer covalently grafted to the surface of a multiwalled carbon nanotube (MWCNT) [79]. At first, a trithiocarbonate-based chain transfer agent was attached to the surface of the MWCNT. Then, the block copolymer poly-*N*-vinyl-1,2,4-triazole-b-poly-*N*-vinyl imidazole (pNVT-b-pNVI) was grown on the functionalized MWCNT surface using the SI-RAFT method. The resulting pNVT-b-pNVI-g-MWCNT was used as a nanofiller in an oxypolybenzimidazole membrane. As a result, a unique homogeneous nanocomposite membrane was obtained, which showed a tensile stress of 1.8 MPa, a strain of 176% at break, and a high proton conductivity of 0.164 S/cm at 180 °C, after doping with phosphoric acid. This significant increase in conductivity is attributed to the proton-conducting nanochannel pathway generated along the block copolymer-g-MWCNT surface [79].

## 5. Conclusions

The research results presented in the peer-reviewed scientific articles indicate that 1*H*-1,2,4-triazole and homopolymers and copolymers of 1-vinyl-1,2,4-triazole have unique physicochemical properties that contribute to a significant improvement in film-forming, thermal, and proton-conducting properties of the polymeric materials based on them. The presence of single–double alternating bonds and three nitrogen atoms in triazole rings ensures effective modification of polymer membranes by various acids and promotes an increase in the activity of proton transfer due to the coordination interaction of triazole fragments with the acid and the formation of low-barrier hydrogen bonds. The maximum values of proton conductivity in the anhydrous state for membranes based on (co)polymers of 1-vinyl-1,2,4-triazole are 10^−1^–10^−2^ S/cm at 120–150 °C, which indicates the prospects for their use and for the development of efficient proton-conducting membranes. However, the main attention of researchers focuses only on the methods of formation of polymer films and the study of their spectroscopic, morphological, and proton-conducting properties. Considering the uniqueness of polymers of 1,2,4-triazole, it is advisable to conduct comprehensive studies on the basic properties necessary for the formation of proton-conducting membranes, such as proton conductivity, mechanical strength, electrochemical stability, ion-exchange capacity, swelling, water absorption, and permeability for fuel.

## Figures and Tables

**Table 1:** Main characteristics of membranes containing 1*H*-1,2,4-triazole in the anhydrous state.

Sample Name	Temperature, °C	Max. Proton Conductivity, S/cm	Reference
1*H*-1,2,4-triazole	115 120	1.5 × 10^−4^ 1.2 × 10^−3^	[23]
1*H*-1,2,4-triazole and 4-dodecylbenzene-sulfonic acid	120	2.0 × 10^−2^	[23]
1*H*-1,2,4-triazole and sulfonated polysulfone	100 140	1.5 × 10^−3^ 5.0 × 10^−3^	[23]
1,2,4-triazole and alginic acid	100	1.0 × 10^−4^	[25]
Poly(vinylidene flouride)–poly(chloromethyl styrene)—1*H*-1,2,4-triazole and trifilic acid	120 150	1.2 × 10^−2^ 2.0 × 10^−2^	[26]
Poly(methacryloyl-1,2,4-triazole) and trifilic acid	150	8.7 × 10^−4^	[27]
Poly(methacryloyl-3-amino-1,2,4-triazole) and trifilic acid	150	2.0 × 10^−2^	[27]
Poly(vinyl alcohol)—sulfosuccinic acid—3-amino-1,2,4-triazole	140	7.26 × 10^−3^	[28]
Poly(vinyl alcohol)—poly (2-acrylamido-2-methylpropane sulfonic acid)—1,2,4-triazole	150	2.0 × 10^−3^	[29]
Poly(phenylene oxide)—1,2,4-triazole and phosphoric acid	150	1.14 × 10^−1^	[30]
1,2,4-Triazolium methanesulfonate	140 190	1.86 × 10^−2^ 3.65 × 10^−2^	[32]
Nafion—1,2,4-triazolium methanesulfonate	140 180	3.67 × 10^−3^ 1.32 × 10^−2^	[32]

**Table 2:** Main characteristics of proton-conducting membranes based on poly-1-vinyl-1,2,4-triazole: maximum proton conductivity in the anhydrous state (σ), ion exchange capacity (IEC), and water uptake (WU).

Sample Name	T, °C	σ, S/cm	IEC, mmol/g	WU, %	Reference
Poly-1-vinyl-1,2,4-triazole and phosphoric acid	50	1.2 × 10^−4^	–	–	[55]
Poly-1-vinyl-1,2,4-triazole and phosphoric acid	140 150	5.0 × 10^−3^ 4.0 × 10^−3^	–	–	[56]
Poly-1-vinyl-1,2,4-triazole and nitrilotri(methyl triphosphonic acid)	150	8.5 × 10^−4^	–	–	[57]
Poly(1-vinyl-1,2,4-triazole) and nitrilotri (methyl triphosphonic acid)	150	8.5 × 10^−4^	–	–	[58]
Poly(1-vinyl-1,2,4-triazole) and poly(vinylphosphonic acid)	180 * 100	2.5 × 10^−5^ * 2.2 × 10^−2^	–	150–350	[59]
Poly(1-vinyl-1,2,4-triazole) and poly(styrene sulfonic acid)	120 150	3.3 × 10^−2^ 1.5 × 10^−2^	–	–	[60]
Poly(1-vinyl-1,2,4-triazole) and toluenesulfonic acid	110 150	1.2 × 10^−2^ 8.0 × 10^−4^	–	–	[61]
Poly(1-vinyl-1,2,4-triazole) and trifluoromethanesulfonic (triflic) acid	80	1.2 × 10^−2^	–	–	[62]
Poly(1-vinyl-1,2,4-triazole) and poly(2-acrylamido2-methyl-1-propanesulfonic acid)	150 * 100	1.1 × 10^−6^ * 3.0 × 10^−1^	–	150–240	[63]
Poly(1-vinyl-1,2,4-triazole) and polybenzimidazole (blends) and phosphoric acid	160	1.1 × 10^−1^	–	33–43	[64]
Poly(1-vinyl-1,2,4-triazole) and poly(vinyl alcohol) with sulfosuccinic acid	150 * 60	2.5 × 10^−5^ * 2.8 × 10^−3^	–	80–150	[65]
Poly(1-vinyl-1,2,4-triazole) with phenol-2,4-disulfonic acid and poly(vinyl alcohol) cross-linked with oxalic acid	80	6.0 × 10^−2^ 7.3 × 10^−3^ 6.2 × 10^−3^	0.9–3.4	50–200	[66,67]
Poly(1-vinyl-1,2,4-triazole) and sulfonated polysulfone and phosphoric acid	150	3.6 × 10^−4^	3.05	–	[68]
Poly(1-vinyl-1,2,4-triazole) and Nafion (blends)	220 * 25	5.3 × 10^−4^ * 1.0 × 10^−3^	–	48–140	[69]

* After humidification (RH = 50%).

**Table 3:** Main characteristics of proton-conducting membranes based on 1-vinyl-1,2,4-triazole copolymers: maximum proton conductivity in the anhydrous state (σ), ion exchange capacity (IEC), and water uptake (WU).

Sample Name	T, °C	σ, S/cm	IEC, mmol/g	WU, %	Reference
Copolymer based on 1-vinyl-1,2,4-triazole and methyl methacrilate doping with phosphoric acid	25 130	1.5 × 10^−2^ 1.1 × 10^−1^	–	–	[72]
Copolymer based on 1-vinyl-1,2,4-triazole and fluoroalkylmethacrylates doping with phosphoric acid	25 130	2.5 × 10^−2^ 0.8 × 10^−1^	–	–	[70,71,72]
Copolymer based on 1-vinyl-1,2,4-triazole and vinylphophonic acid	120	1.0 × 10^−3^	–	–	[73]
Copolymers based on 1-vinyl-1,2,4-triazole and 2-acrylamido-2-methyl-1-propanesulfonic acid	130	2.0 × 10^−3^	–	–	[74]
Copolymers based on 1-vinyl-1,2,4-triazole and 5-(methacrylamido)tetrazole doping with phosphoric acid	150	1.6 × 10^−2^	–	–	[75]
Copolymers based on 1-vinyl-1,2,4-triazole and diisopropyl-p-vinylbenzyl phosphonate doping with phosphoric acid	150	5.0 × 10^−3^	6.1–7.6		[76]
Copolymers based on 1-vinyl-1,2,4-triazole and poly(vinylidene fluoride) doping with triflic acid	150	6.0 × 10^−3^	–	–	[77]
Block copolymers based on 1-vinyl-1,2,4-triazole, 1-vinyl-1,2,4-triazolium salts and N-vinyl pyrrolidone	90	3.1 × 10^−4^	–	–	[78]
Block copolymer based on poly-N-vinyl-1,2,4-triazole and poly-N-vinyl imidazole on the surface of a multiwalled carbon nanotube	180	1.6 × 10^−1^	–	–	[79]

## Data Availability

The data presented in this study are available upon request from the corresponding authors.
